# Supplementary material for: End Sequence Analysis Toolkit (ESAT) expands the extractable information from single-cell RNA-seq data
Source: Genome Res. 2016 Oct;26(10):1397–410. doi: 10.1101/gr.207902.116 (PMC5052061; doi:10.1101/gr.207902.116)
Supplement: Supplemental Material [file supp_gr.207902.116_Supp_Materials.tar.gz › ESAT-esat_v0.1/doc/html/files.html]

ESAT: File List


ESAT


- Main Page
- Related Pages
- Packages
- Classes
- Files

- File List


All Classes Namespaces Files Functions Variables Enumerator Pages

File List

Here is a list of all files with brief descriptions:

[detail level 1234567]

|  |  |
| --- | --- |
| ▼ java |  |
| ▼ broad |  |
| ▼ core |  |
| ► annotation |  |
| AnnotationFactory.java |  |
| AnnotationFactoryFactory.java |  |
| AnnotationHandler.java |  |
| AnnotationReader.java |  |
| AnnotationReaderFactory.java |  |
| AnnotationUtils.java |  |
| BasicAnnotationReader.java |  |
| BasicGenomicAnnotation.java |  |
| BasicGenomicAnnotationReader.java |  |
| BasicLightweightAnnotation.java |  |
| BasicTwoSubjectAnnotation.java |  |
| BED.java |  |
| BEDGraph.java |  |
| BEDGraphReader.java |  |
| BEDReader.java |  |
| Feature.java |  |
| GenomicAnnotation.java |  |
| GenomicAnnotationFilter.java |  |
| GFF.java |  |
| GFFReader.java |  |
| LightweightGenomicAnnotation.java |  |
| MaximumContiguousSubsequence.java |  |
| RepeatMaskerAnnotation.java |  |
| RepeatMaskerAnnotationReader.java |  |
| ShortBED.java |  |
| ShortBEDReader.java |  |
| TwoSubjectAnnotation.java |  |
| ► datastructures |  |
| GenericRootedTree.java |  |
| IllegalTreeOperationException.java |  |
| Interval.java |  |
| IntervalTree.java |  |
| JCSCache.java |  |
| MatrixWithHeaders.java |  |
| MatrixWithHeaderUtils.java |  |
| Pair.java |  |
| ReversibleIterator.java |  |
| ► error |  |
| ParseException.java |  |
| ► hmm |  |
| BadModelException.java |  |
| MarkovModel.java |  |
| MarkovState.java |  |
| ► math |  |
| ANOVA.java |  |
| CombinationGenerator.java |  |
| ComputeFDR.java |  |
| Distribution.java |  |
| EmpiricalDistribution.java |  |
| FDRDistribution.java |  |
| FisherCombinedProbabilityTest.java |  |
| KSTest.java |  |
| Loess.java |  |
| MannWhitney.java |  |
| MannWhitneyPermutations.java |  |
| MathUtil.java |  |
| PermutationDistribution.java |  |
| ScanStatistics.java |  |
| Statistics.java |  |
| UnfairDie.java |  |
| Wilcox2Sample.java |  |
| ► motif |  |
| ► meme |  |
| AMEResultReader.java |  |
| MemeMotif.java |  |
| MemeResultReader.java |  |
| ► misc |  |
| ComputeConservedMotifs.java |  |
| ScoreMotifInstances.java |  |
| PositionWeightColumn.java |  |
| PositionWeightMatrix.java |  |
| PositionWeightMatrixIO.java |  |
| PWMUtils.java |  |
| SearchException.java |  |
| SequenceMotif.java |  |
| SequenceMotifCluster.java |  |
| ► multiplealignment |  |
| AlignmentExtractor.java |  |
| FastaMultipleAlignmentIO.java |  |
| MAFAlignment.java |  |
| MAFIO.java |  |
| MultipleAlignment.java |  |
| MultipleAlignmentFactory.java |  |
| MultipleAlignmentIO.java |  |
| MultipleAlignmentIOFactory.java |  |
| PhylipInterleavedMultipleAlignmentIO.java |  |
| PhylipSequencialMultipleAlignmentIO.java |  |
| ► overlaputils |  |
| GeneSetIntersect.java |  |
| ► parser |  |
| CommandLineParser.java |  |
| StringParser.java |  |
| ► primer3 |  |
| ComputeMIRScore.java |  |
| ComputeOriginalScore.java |  |
| CrossJunctionPCRDesigner.java |  |
| HairpinKmer.java |  |
| isPCRLike.java |  |
| Primer3Exception.java |  |
| Primer3PickingSummary.java |  |
| Primer3PrimerPickingResult.java |  |
| PrimerUtils.java |  |
| RestrictionEnzyme.java |  |
| ► sequence |  |
| AbstractFastaParser.java |  |
| Extractor.java |  |
| FastaHandler.java |  |
| FastaParser.java |  |
| FastaQualityBinaryIO.java |  |
| FastaQualityHandler.java |  |
| FastaQualityIndex.java |  |
| FastaQualityParser.java |  |
| FastaSequenceIO.java |  |
| Sequence.java |  |
| SequenceRegion.java |  |
| SequenceRegionWindow.java |  |
| SequenceUtils.java |  |
| TranscribedRegions.java |  |
| TranscribedSequenceComposition.java |  |
| WindowSlider.java |  |
| ► siphy |  |
| ► tools |  |
| ► conservation |  |
| AverageBranchLength.java |  |
| BatchSubmitEstimateExon.java |  |
| ComputeKmersForRegion.java |  |
| ComputeMaxPercentileOfElements.java |  |
| ComputePercentageOfKmers.java |  |
| EstimateOmegaPerExon.java |  |
| SplitFile.java |  |
| ConservationUtils.java |  |
| ConservedPWMScanner.java |  |
| EvolutionaryModel.java |  |
| EvolutionaryModelParameters.java |  |
| Fit.java |  |
| PhastconsReader.java |  |
| PiHMM.java |  |
| PositionWeightMatrixModel.java |  |
| ScaledWindowParserListener.java |  |
| StationaryDistributionIO.java |  |
| TreeScaler.java |  |
| TreeScalerIO.java |  |
| UnableToFitException.java |  |
| ► util |  |
| ChromosomeStringComparator.java |  |
| CLUtil.java |  |
| CollapseByIntersection.java |  |
| GMTParser.java |  |
| ParseGCTFile.java |  |
| ▼ pda |  |
| ► annotation |  |
| BEDFileParser.java |  |
| GTFFileParser.java |  |
| Locus.java |  |
| ► countreads |  |
| AnnotationCounts.java |  |
| FastqLibraryStats.java |  |
| ► datastructures |  |
| Alignments.java |  |
| AlignmentsReader.java |  |
| LocationAwareMatrix.java |  |
| ► differentialExpression |  |
| BatchNorm.java |  |
| CisVsTrans.java |  |
| CollapseProjections.java |  |
| ConvertToDiscreteMatrix.java |  |
| DifferentialExpression.java |  |
| DifferentialScoring.java |  |
| FixedControlDifferentialExpression.java |  |
| FoldSignificance.java |  |
| FWERDistribution.java |  |
| GrabProbes.java |  |
| GSAProjection.java |  |
| MakePositionalGeneSets.java |  |
| MergeGSEAOutputIntoMatrix.java |  |
| Permutations.java |  |
| RunAllGSEABatch.java |  |
| RunDifferentialExpression.java |  |
| RunGSEA.java |  |
| RunTimeSeries.java |  |
| SplitByName.java |  |
| ► enrichment |  |
| EnrichmentMetric.java |  |
| EnrichmentUtils.java |  |
| ► feature |  |
| ► genome |  |
| Chromosome.java |  |
| DirectoryInstalledGenomeAssembly.java |  |
| ► gene |  |
| functionalAnnotation.java |  |
| GeneTools.java |  |
| GeneWithIsoforms.java |  |
| NeighborAnalysis.java |  |
| NeighborsNullModel.java |  |
| transcriptsNullModel.java |  |
| ► geneexpression |  |
| ► agilent |  |
| ► analysis |  |
| ProcessDataset.java |  |
| AgilentArrayStats.java |  |
| AgilentProbe.java |  |
| AgilentToGCT.java |  |
| AgilentUtils.java |  |
| ClassifyAgilentLincProbes.java |  |
| CollapseProbesByMinPValue.java |  |
| CorrelationAgilentNanostring.java |  |
| CorrelationBetweenReplicates.java |  |
| ExtractSamplesAndLabel.java |  |
| FilterMatrixByMinCutoff.java |  |
| FindProbesForGeneSet.java |  |
| MakeCLSFiles.java |  |
| MakeCLSFromDescription.java |  |
| QCAgilentResults.java |  |
| ► clustering |  |
| AbsolutePearsonFunction.java |  |
| Cluster.java |  |
| ClusterDistanceFunction.java |  |
| ComputeCorrelationOfReplicates.java |  |
| CorrelationMatrix.java |  |
| EuclideanDistance.java |  |
| HierarchicalClustering.java |  |
| Kmeans.java |  |
| PearsonDistance.java |  |
| RankOrder.java |  |
| ComputeHypergeomtricEnrichment.java |  |
| CountNums.java |  |
| ExpressionExperimentInfo.java |  |
| FilterGCT.java |  |
| FilterGCTFile.java |  |
| geneExpUtils.java |  |
| GlobalStats.java |  |
| GrabSpecificColumns.java |  |
| PaGEJava.java |  |
| PreprocessDataSet.java |  |
| ScoreDiffGenes.java |  |
| ► rap |  |
| ► misc |  |
| FindEnrichedDepletedRegions.java |  |
| ► rnai |  |
| ExtractSequence.java |  |
| RNAiGeneAnnotation.java |  |
| ► samtools |  |
| FilterFastqReads.java |  |
| FilterPairedReadsByMAQ.java |  |
| PairedEndReadIterator.java |  |
| PairedEndTDF.java |  |
| SAMFragmentWriter.java |  |
| SAMPairedEndFileReader.java |  |
| TrimFastqReads.java |  |
| WrappedIterator.java |  |
| ► seq |  |
| ► alignment |  |
| AlignmentUtils.java |  |
| StoredAlignmentStats.java |  |
| ► chip |  |
| ComputeEnrichments.java |  |
| FindDiffrentialEnrichedRegions.java |  |
| PeakFilter.java |  |
| ► clip |  |
| FastaExtendPeaks.java |  |
| GenomeSpaceSampleData.java |  |
| Pileup.java |  |
| SampleData.java |  |
| ► fastq |  |
| FastqParser.java |  |
| FastqSequence.java |  |
| FastqSplitterByBarcode.java |  |
| SplitAndTrimIndex.java |  |
| SplitFastqByIndex.java |  |
| ► graph |  |
| ChromosomeWithBubblesJGraphT.java |  |
| Path.java |  |
| ► protection |  |
| BatchedMultiSampleScanPeakCaller.java |  |
| GenomeSpaceSampleData.java |  |
| MultiSampleScanPeakCaller.java |  |
| ReadSimulator.java |  |
| ReadSimulator2.java |  |
| SampleData.java |  |
| TwoSampleScanSkellamPeakCaller.java |  |
| WindowScores.java |  |
| ► rap |  |
| ► rna |  |
| CollectRnaSeqMetrics.java |  |
| CountReads.java |  |
| TranscriptomeCommandLineProgram.java |  |
| TranscriptomeScoringProgram.java |  |
| BuildRatioNullDistribution.java |  |
| CalculateCoverageStats.java |  |
| CollectAnnotationEnrichments.java |  |
| CountProbes.java |  |
| CountReads.java |  |
| DistanceToBED.java |  |
| GCContent.java |  |
| GenomeCommandLineProgram.java |  |
| GenomeScoringProgram.java |  |
| PlotAggregateRegions.java |  |
| RAPPeakCaller.java |  |
| RatioPermutationPeakCaller.java |  |
| ShuffleBAM.java |  |
| ShuffleBED.java |  |
| SkellamKLPeakCaller.java |  |
| SkellamScore.java |  |
| SlideAndCalculate.java |  |
| SlideAndCount.java |  |
| UnmarkDuplicates.java |  |
| ► segmentation |  |
| ► utils |  |
| ReconstructionComparer.java |  |
| AlignmentDataModel.java |  |
| AlignmentDataModelStats.java |  |
| ContinuousDataAlignmentModel.java |  |
| DefineSourceAndSinkFromPairedEnds.java |  |
| GeneCounts.java |  |
| GenericAlignmentDataModel.java |  |
| GeneScore.java |  |
| GenomeWithGaps.java |  |
| GenomeWithGaps2.java |  |
| GlobalSpliceFilter.java |  |
| PrecomputeDataAlignmentStats.java |  |
| ReadCountNormalizer.java |  |
| ReadFilter.java |  |
| WrappedIGVIterator.java |  |
| ► tools |  |
| PSLXToSAM.java |  |
| ▼ projection |  |
| ► gsa |  |
| DefineGeneSignatures.java |  |
| ExtractGenes.java |  |
| GeneSetEnrichment.java |  |
| GSAScoring.java |  |
| GsaUtils.java |  |
| ▼ jaligner |  |
| ▼ formats |  |
| CLUSTAL.java |  |
| FASTA.java |  |
| Format.java |  |
| FormatFactory.java |  |
| Pair.java |  |
| ▼ matrix |  |
| MatricesCompartor.java |  |
| Matrix.java |  |
| MatrixGenerator.java |  |
| MatrixLoaderException.java |  |
| ▼ util |  |
| Commons.java |  |
| SequenceParser.java |  |
| SequenceParserException.java |  |
| Alignment.java |  |
| Cell.java |  |
| Directions.java |  |
| Markups.java |  |
| NeedlemanWunsch.java |  |
| NeedlemanWunschGotoh.java |  |
| Sequence.java |  |
| SmithWatermanGotoh.java |  |
| TracebackNode.java |  |
| ▼ Jama |  |
| ▼ util |  |
| Maths.java |  |
| CholeskyDecomposition.java |  |
| EigenvalueDecomposition.java |  |
| LUDecomposition.java |  |
| Matrix.java |  |
| QRDecomposition.java |  |
| SingularValueDecomposition.java |  |
| ▼ umms |  |
| ▼ core |  |
| ► alignment |  |
| AbstractPairedEndAlignment.java |  |
| Alignment.java |  |
| AlignmentPair.java |  |
| ChromosomeInconsistencyException.java |  |
| FeatureSequenceAlignment.java |  |
| FragmentAlignment.java |  |
| PairedEndAlignmentFactory.java |  |
| PairedReadAlignment.java |  |
| SingleEndAlignment.java |  |
| SmatchLike.java |  |
| SmithWatermanAlignment.java |  |
| ► annotation |  |
| ► filter |  |
| FullyContainedFilter.java |  |
| OverlapFilter.java |  |
| AbstractAnnotation.java |  |
| AbstractAnnotationCollection.java |  |
| Annotation.java |  |
| AnnotationCollection.java |  |
| AnnotationFileReader.java |  |
| AnnotationList.java |  |
| BasicAnnotation.java |  |
| CompoundInterval.java |  |
| Gene.java |  |
| PSLRecord.java |  |
| SingleInterval.java |  |
| ► coordinatesystem |  |
| AnnotationOutOfBoundsException.java |  |
| CoordinateSpace.java |  |
| GenomicSpace.java |  |
| MaskedGenomicSpace.java |  |
| PermutationNotFoundException.java |  |
| ShuffledIterator.java |  |
| TranscriptInGenomicSpace.java |  |
| TranscriptomeSpace.java |  |
| ► exception |  |
| IncompleteMethodImplementationException.java |  |
| RuntimeIOException.java |  |
| ► feature |  |
| GeneWindow.java |  |
| GenomeWindow.java |  |
| Window.java |  |
| ► general |  |
| CloseableFilterIterator.java |  |
| Predicates.java |  |
| TabbedReader.java |  |
| ► model |  |
| ► score |  |
| AbstractListScore.java |  |
| BasicScanStatisticScore.java |  |
| BinomialEnrichmentScore.java |  |
| CountScore.java |  |
| CoverageScore.java |  |
| EndInWindowCountScore.java |  |
| LengthScore.java |  |
| MultiScore.java |  |
| NewRatioScore.java |  |
| PoissonEnrichmentScore.java |  |
| RatioScore.java |  |
| ScanStatisticScore.java |  |
| StartInWindowCountScore.java |  |
| SumScore.java |  |
| WindowAllScore.java |  |
| WindowProcessor.java |  |
| WindowScore.java |  |
| WindowScoreIterator.java |  |
| AlignmentModel.java |  |
| JCSAlignmentModel.java |  |
| ► readers |  |
| PairedEndReader.java |  |
| ► readFilters |  |
| GenomicSpanFilter.java |  |
| MappingQualityFilter.java |  |
| PairedAndProperFilter.java |  |
| PairedEndFilter.java |  |
| PCRDuplicateFilter.java |  |
| ReadEndsInFilter.java |  |
| ReadStartsInFilter.java |  |
| ReadsToReconstructFilter.java |  |
| SameOrientationFilter.java |  |
| SplicedReadFilter.java |  |
| UniqueMappedReadsFilter.java |  |
| ► scripture |  |
| ► statistics |  |
| ConnectDisconnectedTranscripts.java |  |
| BuildScriptureCoordinateSpace.java |  |
| OrientedChromosomeTranscriptGraph.java |  |
| ► utils |  |
| AnnotationUtils.java |  |
| ► writers |  |
| PairedEndWriter.java |  |
| ▼ esat |  |
| CollapsedGeneLengths.java |  |
| ESAT.java |  |
| ESATDistanceCalculator.java |  |


---

Generated on Fri Aug 15 2014 15:11:17 for ESAT by  

 1.8.7
